# Effect of Phosphorus Fertilization on the Growth, Photosynthesis, Nitrogen Fixation, Mineral Accumulation, Seed Yield, and Seed Quality of a Soybean Low-Phytate Line

**DOI:** 10.3390/plants8050119

**Published:** 2019-05-08

**Authors:** Nisar Ahmad Taliman, Qin Dong, Kohei Echigo, Victor Raboy, Hirofumi Saneoka

**Affiliations:** 1 Graduate School of Biosphere Science, Hiroshima University, 1-4-4 Kagamiyama, Higashi-Hiroshima 739-8528, Japan; nisarahmad_kandahar@yahoo.com (N.A.T.); d170372@hiroshima-u.ac.jp (Q.D.); echigo-kohei@hiroshima-u.ac.jp (K.E.); 2 USDA-ARS, Small Grains and Potato Research Unit, 1600 South 2700 West, Aberdeen, ID 83210, USA; vraboy@pdx.edu

**Keywords:** low-phytate soybean, microelement, phosphorus, phytic acid, seed yield, seed quality

## Abstract

Crop seed phosphorus (P) is primarily stored in the form of phytate, which is generally indigestible by monogastric animals. Low-phytate soybean lines have been developed to solve various problems related to seed phytate. There is little information available on the effects of P fertilization on productivity, physiological characteristics, and seed yield and quality in low-phytate soybeans. To address this knowledge gap, studies were conducted with a low-phytate line and two normal-phytate cultivars from western Japan when grown under high- and low-P fertilization. The whole plant dry weight, leaf photosynthesis, dinitrogen fixation, and nodule dry weight at the flowering stage were higher in the higher P application level, but were not different between the low-phytate line and normal-phytate cultivars. As expected, seed yield was higher in the higher level of P application for all lines. Notably, it was higher in the low-phytate line as compared with the normal-phytate cultivars at both levels of fertilizer P. The total P concentration in the seeds of the low-phytate line was the same as that of the normal-phytate cultivars, but the phytate P concentration in the low-phytate line was about 50% less than that of the normal-phytate cultivars. As a result the molar ratio of phytic acid to Zn, Fe, Mn, and Cu in seed were also significantly lower in the low-phytate line. From these results, it can be concluded that growth after germination, leaf photosynthesis, nitrogen fixation, yield and seed quality were not less in the low-phytate soybean line as compared with two unrelated normal-phytate cultivars currently grown in Japan, and that low-phytate soybeans may improve the bioavailability of microelements.

## 1. Introduction

Phosphorus (P) is one of the most essential macro elements, along with nitrogen, required by plants to grow. Adequate P fertilization is essential for effective crop production to attain optimum yields. However, excessive P fertilization increases the risk of P losses to surface and ground waters, with detrimental effects on aquatic ecosystems through eutrophication [1,2]. Phosphorus absorbed by plants is translocated from the roots and leaves to the seeds during seed development, where phytic acid (phytate; myo-inositol 1, 2, 3, 4, 5, 6-hexakisphosphate) is synthesized [3,4]. Phytate accounts for up to 80% of the total P concentration in seeds and its breakdown during germination supplies P and other cations needed for early seedling growth. However, phytate is poorly digested by non-ruminants such as pigs, chickens, and humans, which don’t express phytase, an enzyme that degrades phytate [2,5]. To provide the nutritional requirements of P for animals, and thereby ensure optimal productivity, feeds are supplemented with inorganic phosphorus (inorganic P). Therefore, poultry and chicken manure contain a large amount of phytate-derived P. When these manures are applied to fields, inorganic P is released by the phytase expressed by microorganisms in the soil. If this P is not absorbed by plants, a large amount of P flows into rivers and lakes through rainwater. In addition, dietary phytate chelates divalent cations, including iron (Fe), zinc (Zn), copper (Cu), manganese (Mn), magnesium (Mg), and calcium (Ca), and this reduces the bioavailability and utilization of these essential nutrients [6,7,8].

One approach to these phytate-related problems that increases the bioavailability of P and minerals in seeds is to breed low-phytate crops. Low-phytate lines have been isolated from various agronomic plants such as major cereal crops such as barley (*Hordeum vulgare* L.) [9,10], rice (*Oryza saviva* L.) [11], maize (*Zea mays* L.) [12], wheat (*Triticum aestivum* L.) [13], soybean (*Glycine max* (L.) Merr.) [14], and common bean (*Phaseolus vulgaris* L.) [15,16]. Compared with normal-phytate lines, with one known exception [10] these low-phytate lines have a total P concentration that is no different from normal-phytate lines. They have a 40 to 80% reduction in phytate P, resulting in up to an 80% increase in inorganic P (available P) for animals. From animal feeding experiments using low-phytate lines, it has been confirmed that the P-utilization rates of animals is improved, and the amount of P excreted is decreased [1].

The phytate concentration of seed depends both on the crop variety or its genetics and environmental factors, especially P fertilization. It has been reported that the phytate concentration of seed gradually increases and is positively correlated with applied P levels in soybean [17], oat [18], and maize [19]. However, there have been relatively few reports of analyses of the productivity and seed quality of low-phytate lines grown under varying P fertilization levels [10,20]. Therefore in this study we compared the yield, mineral and phytate concentration of the seeds, and physiological factors such as nitrogen fixation, between a soybean low-phytate line (hereafter referred to as LP) descended (F_10_ and F_11_) from a cross of the normal-phytate Japanese cv. Tanbakuro and the low-phytate line CX1834 [14,21], and two normal-phytate soybean cultivars Enrei and Akimaro, when grown under two different levels of nutrient P fertilization. We asked if phytate accumulation in the seeds of the low-phytate soybean line in response to P fertilization differs from that observed in normal-phytate cultivars and whether a low P fertilizer condition differentially affects seed yield and quality, as well as plant growth and physiological factors such as photosynthesis and nitrogen fixation.

## 2. Results

### 2.1. Biomass Production at the Flowering Stage

**Figure 1:**
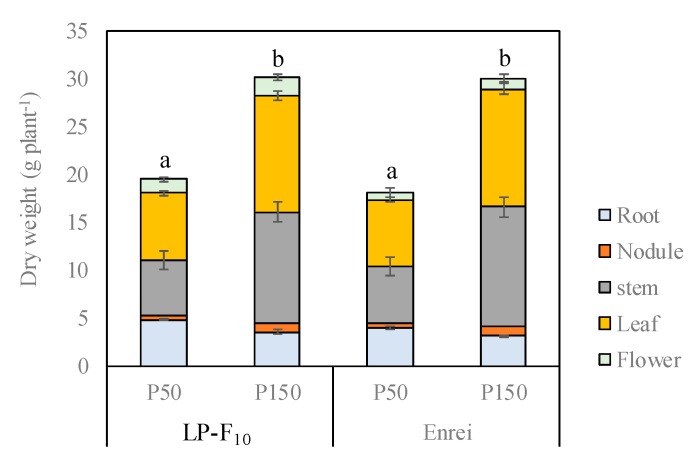
Effect of phosphorus fertilization on whole plant dry weight of the LP-F_10_ progeny and the normal-phytate cv. Enrei (Experiment 1). The same letter indicates no significant difference (*p* ≤ 0.05).

**Figure 2:**
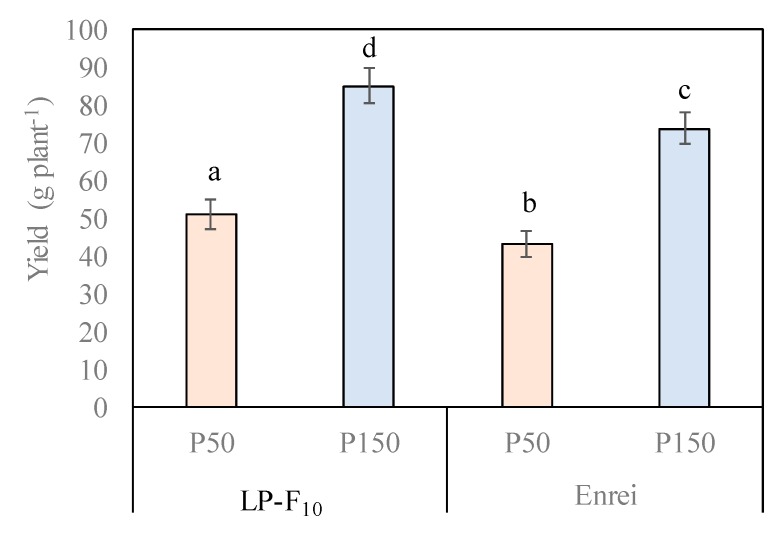
Effect of phosphorus fertilization on seed yield in LP-F_10_ and the normal-phytate cv. Enrei in Experiment 1. The same letter indicates no significant difference (*p* ≤ 0.05).

**Figure 3:**
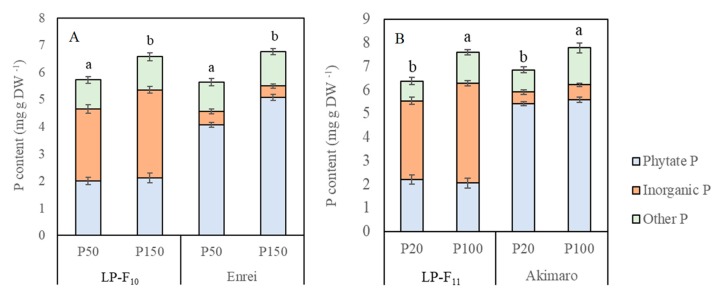
Effect of phosphorus fertilization on seed total P, phytate P and inorganic P concentrations in LP lines and normal-phytate soybean cultivars of soybean in Experiment 1 (**A**) and Experiment 2 (**B**). The same letter indicates no significant difference (*p* ≤ 0.05).

In Experiment 1 we compared the LP-F_10_ progeny with the cultivar Enrei (Figure 1, Figure 2 and Figure 3 and Table 1). The whole plant weight of both the LP-F_10_ line and Enrei was significantly affected by the higher P application at the flowering stage (Figure 1). The whole plant weight in both the LP line and normal-phytate cultivar in the P150 treatment was 1.5 to 1.6 times higher than that in the P50 treatment, respectively. However, there was no difference in the whole plant weight between the LP line and normal-phytate cv. Enrei within the same P treatment.

### 2.2. Photosynthesis and Dinitrogen Fixation Activity at the Flowering Stage

In Experiment 1 the photosynthetic rate and dinitrogen fixation activity in LP-F_10_ and cv. Enrei were 1.3 and 1.5 times higher (respectively) in the P150 treatment than in the P50 treatment, but there was no substantial differences between LP-F_10_ and Enrei within a given P treatment (Table 1). The nodule number was higher in the higher P application level for both LP-F_10_ and Enrei however the nodule number of LP-F_10_ was higher than that of Enrei when grown under the P50 treatment. The specific nodule activity in both LP-F_10_ and Enrei, obtained by dividing the nitrogen fixation rate by the root nodule weight, was greater in the P50 treatment than in the P150 treatment. No significant differences were observed in specific nodule activity of LP-F_10_ as compared with Enrei within a given P treatment.

### 2.3. Seed Yield and Yield Components

In Experiment 1, seed yield of both LP-F_10_ and cv. Enrei was 1.7 times higher in the P150 treatment as compared with the P20 treatment (Figure 2). In Experiment 2 seed yield was 2.4 times higher in LP-F_11_ and 2.2 times higher in Akimaro in the P100 treatment as compared to the P50 treatment (Table 2). Importantly, in both experiments, in a given P treatment level the seed yield of the LP line was significantly (*p* ≤ 0.05) higher than the normal-phytate line, by 16% to 34%. Pod number data was not collected in Experiment 1. In Experiment 2, the number of pods per plant was significantly higher (*p* ≤ 0.05) in the P100 treatment as compared with the P20 treatment in both the LP-F_11_ line (2.1 times higher) and Akimaro (2.3 times higher, Table 2). Of importance here is that the pod number per plant of the LP-F_11_ line was higher than that of Akimaro in both P treatments; 1.41 and 1.28 times higher in the P20 and P100 treatments, respectively, but only significantly different, *p* ≤ 0.05 in the P100 treatment. P treatment did not greatly affect 100 seed weight in either line or cultivar (Table 2). The 100 seed weight was about 1.6 times higher in LP-F_11_ than in Akimaro in both P treatments, but only significantly greater (*p* =≤ 0.05) in the P20 treatment.

### 2.4. Seed Total P, Phytate P and Inorganic P Concentration

In Experiment 1, the seed total P concentration in both LP-F_10_ and cv. Enrei was ~5–6 mg g^−1^ DW in the P50 treatment and ~6–7 mg/g DW in the P 150 treatment but did not differ between LP-F_10_ and Enrei (Figure 3A). The percentage of total P represented by phytate P in LP-F_10_ (~32–35%) was about half that of Enrei (~72–75%), and the percentage of total P represented by inorganic P in LP-F_10_ (~46–49%) was ~5–6 times greater than that of Enrei (~6–9%). Seed phytate P concentration within in a given line or cultivar was not consistently impacted by P treatment, whereas inorganic P was 15% higher in LP lines grown with P150 as compared with P50. Overall, similar results were observed in Experiment 2 (Figure 3).

### 2.5. Seed Crude Protein, Lipid and Mineral Concentrations

Data for seed crude protein, lipid and mineral concentrations was only collected in Experiment 2 (Table 3). Seed crude protein and lipid concentrations in both LP-F_11_ and cv. Akimaro were about 10% higher in the P100 versus the P20 P treatments (Table 3). The seed crude protein concentration in both P treatments was 3% to 12% higher in LP-F_11_ than in the Akimaro; however, the lipid concentration was 5% to 12% higher in Akimaro that in LP-F_11_.

The K and Ca concentrations in the seeds of both LP-F_11_ and cv. Akimaro were 13% to 37% higher in the P100 treatment as compared with the P20 treatment (Table 3). While the seed Mg concentration in LP-F_11_ was not different between the P20 and P100 treatments, the seed Mg concentration in Akimaro was 15% higher in the higher P application level. Seed K concentration in the P20 treatment was 17% higher in LP-F_11_ than in Akimaro, but no differences between lines were observed in the P100 treatment. Statistically significant differences in seed Ca and Mg concentrations were not observed between LP-F_11_ and Akimaro at a given level of P application. In contrast, the seed Zn, Mn, and Cu concentrations in both LP-F_11_ and Akimaro were 20% to 30% lower in the P100 fertilization level as compared with the P20 level, probably due to a dilution effect resulting from the higher seed yields at the higher P fertilization rate. With the exception of seed Fe levels in the P100 treatment and Cu at the P100 level, these element’s concentrations were 11% to 43% higher in LP-F_11_ seed than in Akimaro in both P application levels. For both LP-F_11_ and Akimaro there was little difference in the molar ratio of phytic acid to Zn, Fe or Mn between the two levels of P fertilization. However for Cu, the phytic acid:Cu molar ratio observed for the P100 treatment was 1.68 and 1.73 times as great in the P100 versus P20 fertilization levels for LP-F_11_ and Akimaro, respectively. Of importance for this study, due to Akimaro’s 2.5 to 2.7-times higher level of phytic acid as compared with LP-F_11_, phytic acid molar ratios to Zn, Fe, Mn, and Cu were 2.5 to 3.5-times higher in Akimaro seed as compared with LP-F_11_ seed across both P fertilization treatments (Table 3).

## 3. Discussion

Many studies on the influence of P application on the growth, photosynthesis, nitrogen fixation, and yield of soybean and other crop species have been reported [17,22,23,24,25,26]. Most of these studies were carried out with crop cultivars that have “standard” (non-mutant) levels of seed phytic acid. In contrast relatively few such studies have been conducted with low-phytate variants of crops species [10,20] including one with low-phytate soybean lines [20] which will be discussed below. The proportion of phytate P to total P concentration in the seed of normal soybean cultivars is about 70–80%, but that of the low-phytate lines that we bred is about 30–35%. It has been reported that many low-phytate mutants impact plant growth and productivity and have decreased yield as compared with sibling normal-phytate/wild-type lines [27,28,29]. However in the case of soybean lines developed from crosses using the same low-phytate parent used here, CX1834 [14], previous studies demonstrated that while yields of low-phytate progeny were 85% to 90% of sibling normal-phytate lines, the yield loss was largely due to negative impacts on germination and emergence that led to reduced stand establishment, rather than impacts on plant growth and productivity subsequent to emergence and germination [30,31]. Therefore, in this study, we further investigated whether in the low-phytate lines that we bred, there was an effect on the growth, photosynthesis, nitrogen fixation, seed yield, and seed quality subsequent to germination and emergence. In addition, it has been reported that the phytate concentration of the seed of normal-phytate cultivars is increased by P application [17,19], and that there is a highly positive correlation between total P and phytate in seeds [17]. Therefore, we investigated whether the phytate concentration of seed increases with a higher P application level even in a low-phytate line.

Highly significant effects of the P application level on the plant growth, leaf photosynthesis, biological nitrogen fixation of nodules, and yield of seed were observed in this study. With higher application of P, the whole plant dry weight was markedly higher in Experiment 1 (Figure 1). However, while an increase in whole plant dry weight in the P 150 treatment compared to the P 50 treatment was observed, there were no differences in whole plant dry weight between the low-phytate line and normal-phytate cultivar in either P treatment. These results suggest that the decrease of phytate in the seeds of the low-phytate line did not substantially affect the growth of plants subsequent to germination at any P application level.

At the low P level, the photosynthetic rate and dinitrogen fixation activity of both the low-phytate line and normal phytate cultivar were less than those of the higher P application level (Table 1). There are many reports that photosynthesis increases with P application. Wang et al. [26] reported that the activity of Rubisco, which is a key enzyme of photosynthesis, and the protein concentration of leaves were higher in a high P application than in a low P application. Furthermore, Singh and Reddy [32] also reported that Rubisco activity, RUBP regeneration, and maximum quantum yield related to the photochemical system were decreased under a P-deficient condition. Dinitrogen fixation activity was also increased by P application. It is assumed that the increase in dinitrogen fixing activity by P application is due to an increase in nodule weight (Table 1). In any case, photosynthesis and dinitrogen fixation were lower in the low P level, but the reduction was not significantly different between the low-phytate line and normal-phytate cultivar. These results indicate that both seed phytate concentration and low-phytate genotype, at least those low-phytate lines derived from crosses using the CX-1834 source used here, have little impact on photosynthesis and dinitrogen fixation in soybeans at the growth stages following germination.

In this study, yield and yield components of both the low-phytate line and normal-phytate cultivar were significantly affected by P application. As expected, the yield of both the low-phytate line and normal-phytate cultivar was higher in the high P level than in the low P level. However, the low-phytate line had significantly higher seed yields than the normal-phytate cultivar in all the treatments (Figure 2 and Table 1). These differences were mainly attributed to the number of productive pods and 100 seed weight (Table 1).

Since comparisons here were made between low-phytate soybean lines obtained from the cross of cv. Tanbakuro and the low-phytate line CX1834 [14], and the unrelated normal-phytate cultivars Enrei and Akimaro, and not between the low-phytate line and a sibling normal-phytate/wild-type line derived from the same Tanbakuro by CX1834 cross, we cannot attribute any observed differences in yield or any trait to allelic differences in genes conditioning the low-phytate trait. We can only conclude that on a single-plant basis and not taking into account any effect on germination and emergence, yield and other traits of these specific low-phytate soybean progeny was observed to be equal to or better than either of these unrelated normal-phytate cultivars currently grown in Japan.

A previous study of several low-phytate soybean lines grown under varying levels of P supply in hydroponics reported that two such lines were more productive in terms of seed yield at given level of nutrient P than were the non-mutant, normal-phytic acid parental line, even when these lines were grown under a very growth-limiting low-nutrient P level [20]. However, one other low-phytate soybean line evaluated in this earlier study displayed reduced seed yield when grown under limiting nutrient P. Therefore one can conclude from the present and earlier studies that in soybean the impact of the low-phytate trait on growth and yield when grown under varying levels of nutrient P is variable, depending on genotype and level of nutrient P, but that some low-phytate soybean lines appear to be as productive if not more productive than normal-phytate lines.

In the two closely related food legume species soybean and common bean, there are two copies of the gene perturbed in the mutant studied here, a member of the MRP gene family that encodes an ABC transporter specific to phytic acid transport [16,33,34]. The low-phytate trait in the original low-phytate soybean parental line CX1834 line [14] that was used as the trait donor in breeding the low-phytate line studied here [21], was found to be conditioned by mutations in both copies of this particular MRP gene in the soybean genome [33]. In contrast, in the common bean low-phytate mutants that are conditioned by mutations in its MRP ortholog, the low-phytate trait is the result of a mutation in only one of the two MRP duplications, and there is little or no negative impact on germination, emergence or subsequent plant growth, performance or yield [15,16]. It was hypothesized that this lack of negative impact or pleiotropic effect is due to a buffering effect resulting from functional complementation provided by the second wild-type duplication of the gene [16,34]. Therefore it is possible that when transferring the trait from CX1834 into the parental background cv. Tanbakuro used to breed the of the low-phytate line studied here, a similar buffering effect was obtained, either provided by variant alleles of one of the two MRP orthologs, or perhaps by other components of the Tanbakuro genome. However the most likely explanation for the good growth and performance of the low-phytate soybean lines studied here is that problems with performance of this specific genotype are probably due to negative impacts on germination and emergence, rather than to negative impacts on subsequent growth and performance [29,30,31].

Highly significant effects of higher P application on the total P and phytate P concentrations of the seeds were found in both Experiment 1 (Figure 3A) and Experiment 2 (Figure 3B). While total P concentration was higher in the higher level of P application there was little difference in total P between the low-phytate line and normal-phytate cultivar. However the substantial effects of P fertilization on phytate P and inorganic P concentrations differed between low-phytate lines and normal-phytate cultivars. For example, in Experiment 1 the phytate P concentration in the normal-phytate cultivar was 4.09 mg g^−1^ at the 50 P application level and 5.26 mg g^−1^ at the 150 P level. However, in the low-phytate line, phytate P concentrations were similar at both P fertilization levels, about 2.0 mg g^−1^ dry weight. Similar results were obtained in Experiment 2 (Figure 3B). In the low-phytate line, inorganic P increases occurred instead of increasing phytate P in response to increasing nutrient P supply. As an outcome of this, the proportion of phytic acid P to total P concentration was reduced with increased P fertilization in the low-phytate lines but not greatly affected by the level of P application in the normal phytate lines (Figure 3). These results support the finding of previous studies that indicate that the accumulation and synthesis of phytic acid in seed is highly dependent on the soil P level [35]. A highly significant positive relationship between P supply and phytic acid P in seeds has been observed in soybean [17] and maize [19]. The results of this study agree with that of Oltmans et al. [36] who reported that the mean total P of a similar low-phytate line and its sibling normal-phytate lines was not significantly different, but the mean phytate P, inorganic P, and other P concentrations were significantly different.

Lipids and protein are important factors in seed quality. There are some reports that the lipid and protein concentrations of seed were increased or decreased by P fertilization, and the influence an lipids and proteins by P application was different depending on the study. For example, in soybean, Abbasi et al. [37] reported that P application increased the oil (lipid) and protein concentrations, but Yi et al. [38] reported that the protein concentration was increased, and oil concentration was decreased with increased P application. Krueger et al. [39] reported that oil (lipid) and protein concentrations were not affected by P fertilization. Bethlenfalvay et al. [40] also found that soybean seed lipid and protein concentrations were not significantly correlated, and there was a highly significant negative correlation between seed P and lipid concentration. In this study, the lipid and protein concentration of seed in both the low-phytate line and normal-phytate cultivar were significantly higher in the higher P application level (Table 3). From this study and previous reports, the influence of P application on the protein and lipid concentration of soybean seed may be different depending on the P application level, soil moisture condition, and characteristics of the cultivar used in the study. In any case, from this study, even when the low-phytate line and normal-phytate cultivar were cultivated under the same P application condition, there was no difference in the lipid and protein concentration of the seed.

The differences in seed K, Ca, and Mg concentrations observed here could be attributed to the differences in seed phytate concentration that result either from differences in P fertilization or low-phytate genotype (Table 3). A similar result was found by Toda et al. [41]: since Ca and Mg ions form salts with phytic acid and as a result diminish its buffering effect, unbound phosphate groups of phytic acid would result from the low concentration of Ca and Mg. In contrast to K, Ca, and Mg, microelements, such as Zn, Fe, Mn, and Cu in the both low-phytate line and normal-phytate cultivar were less in the higher P application level. It is well known that the concentration of these microelements is affected by P fertilization. Naeem et al. [23] reported that the Zn concentration of seed in wheat was decreased by P application and Sánchez-Rodríguez et al. [42] reported the same in wheat and barley. Zhang et al. (2012) [43] also reported that P application significantly decreased Zn and Cu, but had no effect on the Fe and Mn concentrations of wheat seed. Zhang et al. (2017) [44] also reported that total P to Zn, Cu, Fe, and Mn molar ratios in maize seed were increased, which indicates that P application in maize may affect the bioavailability of these minerals. In this study, Zn, Fe, and Mn levels and the phytic acid:mineral molar rations for Zn, Fe and Mn were not greatly affected by differing P fertilization in either the low-phytate line or normal-phytate cultivar (Table 3). However the higher level of phytic acid in the normal-phytate cultivar as compared with the low-phytate line when grown on low or high fertilizer P resulted in much higher (2.5 to 3.5-fold) phytic acid:mineral molar ratios. From this result, it can be suggested that the low-phytate line maintained high bioavailability of these microelements in comparison to the normal-phytate cultivar regardless of P application, and will likely have positive effects on the nutrition of monogastric animals.

## 4. Materials and Methods

### 4.1. Plant Material and Growing Condition

The low-phytate soybean line CX1834, isolated from the mutant originally referred to as M153 [14], was obtained from USDA-ARS, National Small Seeds Germplasm Research Unit, Aberdeen, Idaho. This low-phytate line was used as the pollen parent in a cross with the Japanese commercial cultivar “Tanbakuro” [21]. These two parents were crossed in August 2004 under greenhouse conditions, and ten progeny populations were developed from the F1 seeds. The F1 generation and the parents were grown in a field of the Graduate School of Biosphere Science, Hiroshima University, Higashi-Hiroshima, Japan, during the summer of 2005, and low-phytate lines were selected by measuring the phytate and inorganic and total phosphorus concentrations in seeds after harvesting. In this study, we conducted two experiments using one selected low-phytate line each (F10 line for Experiment 1 and F11 line for Experiment 2) and one normal-phytate cultivar each (Enrei for Experiment 1 and Akimaro for Experiment 2), that are cultivated in western Japan. Both the low-phytate and normal-phytate soybeans were sown in a seed bed containing a soil mixture of granite regosol soil, perlite, peat moss, and nursery soil with compost (2:1:1:1 per volume base). Inoculation of root nodule bacteria (*Bradyrhizobium japonicum*) was performed by mixing a small amount of soil from the previous year’s cultivation into the seedbed. Both experiments were conducted in the greenhouse for prevention of rainfall.

Experiment 1: Both soybean F_10_ line and cv. Enrei were planted in seed beds containing a mixture of soil as above on 3 June, 2016. After 13 days of germination, uniform seedlings were transplanted to a wooden container (30 cm in width, 10.5 m in length, and 18 cm in depth) filled with a mixture of soil. The plant-to-plant space was 20 cm in containers separated by 100 cm distance from each other. Each treatment was separated by inserting a water proof plastic sheet in order to prevent moisture and nutrient movement between the treatments in the container. Before transplanting, the soil mixture was fertilized with a basal fertilization of 100 kg ha^−1^ of K_2_O as potassium sulfate. The soil pH (H_2_O) was adjusted to about 6.0 with dolomitic calcium carbonate. P treatment provided the following two fertilization levels: 50 and 150 kg P_2_O_5_ ha^−1^ as single super phosphate in a randomized complete block design with four replications. At the flowering stage in mid-July, the leaf photosynthetic rate and nitrogen fixation of the nodule were measured. At the full-maturity stage in mid-October, plants were harvested and the stem was separated. The seeds and stem were dried at 80 ℃ in an air-forced oven for more than 3 days, and weighed.

Experiment 2: Both soybean F11 line and cv. Akimaro were planted in seed beds containing a mixture of soil on 11 June, 2017. After 14 days of germination, one seedling each was transplanted to 9 liters pots (25 cm in diameter, and 23 cm in depth) filled with soil mixture. The pot-to-pot distance was 30 cm with no interaction between the plant roots. Before transplanting, as in Experiment 1, the soil mixture was fertilized with a basal fertilization of 100 kg ha^−1^ of K_2_O as potassium sulfate. The soil pH (H_2_O) was adjusted to about 6.0 with dolomitic calcium carbonate. P treatment provided the following two fertilization levels: 20 and 100 kg P_2_O_5_ ha^−1^ as single super phosphate in a randomized complete block design with four replications. The plants were harvested at full-maturity stage in mid-October. After pod and seed numbers were counted and the seeds were weighed, the seeds were oven dried at 70 ℃ for 72 h for analysis of the mineral and oil concentration.

### 4.2. Determination of Leaf Photosynthesis and Dinitrogen Fixation

The dinitrogen fixation activity and leaf photosynthetic rate were determined at flowering stage in Experiment 1. The photosynthetic rate was measured on the upper most fully-expanded leaf using portable infrared gas analyzer (Model LI-6400, Licor Co. Ltd., NE, USA) between 10:30 a.m. and 12:30 p.m. While taking the measurements, the photosynthetic active radiation was adjusted at 1200 µmol m^−2^ S^−1^, the humidity was 65%, the leaf temperature was 28 ℃, and the ambient CO_2_ concentration was 350 mol L^−1^. Dinitrogen fixation was analyzed by measuring the reduction of acetylene to ethylene at flowering time as follows. Intact root systems were excised and gently separated from the soil. The root system and attached nodules were quickly placed in a 1000 mL glass bottle. The bottle was sealed with a rubber stopper and 100 mL of air in the bottle was replaced with acetylene. Samples were incubated at ambient laboratory temperatures. During the incubation periods, 0.3 mL of gas samples were extracted at 10 min and 1 hour and injected into a gas chromatograph (Shimazu GC-14B, Shimazu Co., Kyoto, Japan) fitted with a flame ionization detector to determine the ethylene concentration. Specific nitrogenase activity was calculated by dividing the nitrogenase activity of each sample by the dry weight of the nodules.

### 4.3. Determination of Seed Mineral Concentration

Samples of dried seeds were finely ground using a vibrating sample mill (TI-100, Heiko, Japan) and the mineral concentrations of the seed were measured. Finely ground samples were digested by Sulfuric Acid-Hydrogen Peroxide (H_2_SO_4_–H_2_O_2_), and the K concentration was measured using a flame photometer (ANA 135, Tokyo Photoelectric, Tokyo, Japan). Ca and Mg were measured using an atomic absorption flame emission spectrophotometer (AA-6200, Shimadzu, Japan). The total P was determined using a UV-Spectrophotometer (U-3310, Hitachi Co. Ltd. Tokyo, Japan) following the molybdenum reaction solution method [45]. The total nitrogen was measured using the Kjeldahl method after sample digestion with concentrated H_2_SO_4_ and H_2_O_2_. The crude protein concentration was calculated based on converting the seed nitrogen to protein percentage by multiplying by a conversion factor of 6.25 [46].

### 4.4. Determination of Phytate P and Inorganic P Concentration

Seed phytate P was measured according to the method by Raboy et al. [47], where aliquots of flour were extracted in extraction media (0.2 M HCl: 10% Na_2_SO_4_) overnight at 4 °C with shaking. Extracts were centrifuged, and phytate was obtained as a ferric precipitate and assayed for P calorimetrically using ammonium molybdate reaction reagent. Inorganic phosphorus was extracted in trichloroacetic acid (12.5%) + MgCl_2_ (2 mmol L^−1^) while stirring overnight, and Pi was measured using colorimetric methods [45]. The phytic acid concentration was calculated by multiplying phytic acid P values by 3.55 as described by Raboy and Dickinson [48].

### 4.5. Determination of Zn, Fe and Mn Concentration

Determination the Zn, Fe, and Mn concentration, the seeds were digested by HNO_3_ and H_2_O_2_ (4:1) and measured using an inductively coupled argon atomic emission spectrometer (iCAP 6000, Thermo Fisher Scientific Inc. USA).

### 4.6. Determination of Lipid Concentration

The lipid concentration was measured by chloroform–methanol 2:1 (v/v) according to the method by Folch et al. [49]. For each sample, a mixed solution of chloroform–methanol (2:1) was added to the dried fine seed samples in a vented conical Erlenmeyer flask, and heated at 65℃ for 30 min and boiled for 1 hour. After extraction, the samples were cooled and filtered in an eggplant-type flask using a glass filter with additional mixed solution of chloroform-methanol. The chloroform–methanol solution was evaporated from the sample and were petroleum ether and sodium sulfate were added, the solution was shaken. The samples were centrifuged at 3000 rpm, and the supernatant was transferred to a weighing glass tube and dried at 105℃. After evaporation of the ether, the glass tube was weighed and the lipid concentration calculated.

### 4.7. Statistical Analysis

All the collected data were subjected to analysis of variance using SPSS statistics package, Student Version 19, and means (n = 4) were separated using the Duncan Multiple Range Test at *p* = 0.05.

## 5. Conclusions

It is evident from previous literature that phosphorus fertilizer will significantly increase the whole plant growth, leaf photosynthesis, dinitrogen fixation of nodules, and yield of seed. In this study, we further found no significant differences in these physiological parameters between the low-phytate line and normal-phytate cultivar under any fertilization conditions. While seed crude protein and lipid concentrations were found to be higher in the higher levels of P application in both the low-phytate line and normal-phytate cultivar, seed crude protein concentration was higher in the low-phytate line than in the normal-phytate cultivar. These results suggest that reducing phytic acid in seeds does not affect growth after leaf photosynthesis, dinitrogen fixation of nodules, or final yield of plants following germination and stand establishment. P application had little effect on seed Zn, Fe, and Mn, but increasing P fertilization increased seed Cu by 1.7-fold regardless of genotype or cultivar background. The lower molar ratios of phytic acid to Zn, Fe, Cu, and Mn in seed of the low-phytate soybean lines as compared with the normal-phytate soybean cultivars should enhance the bioavailability of these microelements.

## Figures and Tables

**Table 1:** Effect of phosphorus fertilization on photosynthesis and nitrogen fixation in LP-F_10_ and the normal-phytate cv. Enrei (Experiment 1). The same letter indicates no significant difference (*p* ≤ 0.05).

Line or Cultivar	Treatment	Photosynthetic Rate	Nitrogen Fixation	Nodule Number	Specific Nodule Activity
		(μmol CO_2_ m^−2^ s^−1^)	(μmol C_2_H_4_ plant^−1^ h^−1^)	(number plant^−1^)	(μmol g^−1^ nodule weight h^−1^)
LP-F_10_	P50	14^b^ ± 1	158^b^ ± 9	119^b^ ± 6	310^a^ ± 12
	P150	18^a^ ± 1	227^a^ ± 8	146^a^ ± 5	236^b^ ± 15
Enrei	P50	13^b^ ± 2	151^b^ ± 6	102^c^ ± 3	336^a^ ± 14
	P150	18^a^ ± 2	203^a^ ± 10	138^a^ ± 4	246^b^ ± 16

**Table 2:** Effect of phosphorus fertilization on yield and yield attributes in LP-F_11_ and the normal-phytate cv. Akimaro in Experiment 2. The same letter indicates no significant difference (*p* ≤ 0.05).

Line or Cultivar	Treatment	Yield	Pod Number	100 Seed Weight
		(g plant^−1^)	(number plant^−1^)	(g)
LP-F_11_	P20	42^c^ ± 4	75^c^ ± 2	24^a^ ± 1
	P100	99^a^ ± 4	156^a^ ± 3	26^a^ ± 1
Akimaro	P20	33^d^ ± 4.	53^c^ ± 2	15^b^ ± <1
	P100	74^b^ ± 4.	122^b^ ± 5	16^b^ ± 1

**Table 3:** Effect of phosphorus fertilization on seed quality in the low-phytate LP-F_11_ as compared with the normal-phytate cv. Akimaro soybean in Experiment 2. The same letter indicates no significant difference (*p* ≤ 0.05).

Seed Quality	LP-F_11_		Akimaro	
	P20	P100	P20	P100
Protein (%)	33^b^ ± 2	38.9^a^ ± 1.9	31.9^b^ ± 1.3	34.8^b^ ± 1.5
Lipid (%)	19^c^ ± < 1	23^a^ ± <1	22^b^ ± <1	24^a^ ± <1
K (mg g^−1^)	203^b^ ± 16	229^a^ ± 17	174^c^ ± 15	238^a^ ± 14
Ca (mg g^−1^)	0.97^b^ ± 0.1	1.35^a^ ± 0.2	1.13^b^ ± 0.1	1.49^a^ ± 0.1
Mg (mg g^−1^)	2.2^a^ ± 0.2	2.2^a^ ± 0.3	2.5^a^ ± 0.3	2.9^a^ ± 0.3
Zn (μg g^−1^)	48^a^ ± 3	39^b^ ± 3	39^b^ ± 3	35^c^ ± 3
Fe (μg g^−1^)	51^a^ ± 7	49^a^ ± 6	41^b^ ± 6	49^a^ ± 7
Mn (μg g^−1^)	63^a^ ± 6	50^b^ ± 4	44^bc^ ± 4	38^c^ ± 4
Cu (μg g^−1^)	7.3^a^ ± 0.7	4.1^b^ ± 0.4	6.4^a^ ± 0.6	3.8^b^ ± 0.4
Phytic acid (mg g^−1^)	7.8^b^ ± 04	7.4^b^ ± 0.2	19.2^a^ ± 1	19.8^a^ ± 1
Phytic acid:Zn (Molar ratio)	18.4^c^ ± 0.7	18.0^c^ ± 0.9	45.3^b^ ± 4.2	56.3^a^ ± 3.4
Phytic acid:Fe (Molar ratio)	12.9^b^ ± 0.6	12.8^b^ ± 0.8	39.7^a^ ± 4.2	34.4^a^ ± 3.3
Phytic acid:Mn (Molar ratio)	10.3^c^ ± 0.8	12.4^c^ ± 0.6	36.1^b^ ± 1.6	43.7^a^ ± 2.5
Phytic acid:Cu (Molar ratio)	103.9^d^ ± 6.8	174.5^c^ ± 8.7	291.0^b^ ± 20.5	503.6^a^ ± 18

## References

[1] Raboy V. (2001). Seeds for a better future: ‘low phytate’ seeds help to overcome malnutrition and reduce pollution. Trends Plant Sci..

[2] Vats P., Bhattacharyya M., Banerjee U. (2005). Use of phytases (myo-inositol hexakisphosphstate phosphohydrolases) for combatting environmental pollution: A biological approach. Environ. Sci. Technol..

[3] Lott J.N.A., Ockenden I., Raboy V., Batten G.D. (2000). Phytic acid and phosphorus in crop and fruits: A global estimate. Seed Sci. Res..

[4] Urbano G., López-Jurado M., Aranda P., Vidal-Valverde C., Porres T.J. (2000). The role of phytic acid in legumes: Antinutrient or beneficial function?. J. Physiol. Biochem..

[5] Raboy V. (2009). Approaches and challenges to engineering seed phytate and total phosphorus. Plant Sci..

[6] Erdman J. (1981). Bioavailability of trace minerals from cereals and legumes. Cereal Chem..

[7] Persson H., Türk M., Nyman M., Sandberg A.S. (1998). Binding of Cu^2+^, Zn^2+^, and Cd^2+^ to inositol tri-, tera-, penta-, and hexaphosphates. J. Agric. Food Chem..

[8] Holm P.B., Kristiansen K.N., Pedersen H.B. (2002). Transgenic approaches in commonly consumed cereals to improve iron and zinc concentration and bioavailability. J. Nutr..

[9] Larson S.R., Young K.A., Cook A., Blake T.K., Raboy V. (1998). Linkage mapping of two mutations that reduce phytic acid concentration of barley seed. Theor. Appl. Genet..

[10] Raboy V., Cichy K., Peterson K., Reichman S., Sompong U., Srinives P., Saneoka H. (2014). Barley (*Hordeum vulgare* L.) *Low phytic acid 1-1*: An endosperm-specific, filial determinant of seed total phosphorus. J. Hered..

[11] Larson S.R., Rutger J.N., Young K.A., Raboy V. (2000). Isolation and genetic mapping of a non-lethal rice (*Oryza sativa* L.) low phytic acid 1 mutation. Crop. Sci..

[12] Raboy V., Gerbasi P.F., Young K.A., Stoneberg S.D., Pickett S.G., Bauman A.T., Murthy P.P., Sheridan W.F., Ertl D.S. (2000). Origin and seed phenotype of maize *low phytic acid 1-1* and *low phytic acid 2-1*. Plant Physiol..

[13] Guttieri M., Bowen D., Dorsch J.A., Raboy V., Souza E. (2004). Identification and characterization of low phytic acid wheat. Crop. Sci..

[14] Wilcox J.R., Premachandra G.S., Young K.A., Raboy V. (2000). Isolation of high seed inorganic P, low-phytate soybean mutants. Crop. Sci..

[15] Campion B., Sparvoli F., Doria E., Tagliabue G., Galasso I., Fileppi M., Bollini R., Nielsen E. (2009). Isolation and characterisation of an lpa (low phytic acid) mutant in common bean (*Phaseolus vulgaris* L.). Theor. Appl. Genet..

[16] Cominelli E., Confalonieri M., Carlessi M., Cortinovis G., Daminati M.G., Porch T.G., Losa A., Sparvoli F. (2018). Phytic acid transport in Phaseolus vulgaris: A new low phytic acid mutant in the PvMRP1 gene and study of the PvMRPs promoters in two different plant systems. Plant Sci..

[17] Raboy V., Dickinson D.B. (1993). Phytic acid levels in seeds of *Glycine max* and *G. soja* as influenced by phosphorus status. Crop. Sci..

[18] Miller G.A., Youngs V.L., Oplinger E.S. (1980). Effect of available soil-phosphorus and environment on the phytic acid concentration in oats. Cereal Chem..

[19] Saneoka H., Koba T. (2003). Plant growth and phytic acid accumulation in seed as affected by phosphorus application in maize (*Zea maize* L.). Grassl. Sci..

[20] Kumar V., Singh T.R., Hada A., Jolly M., Ganapathi A., Sachdev A. (2015). Probing phosphorus efficient low phytic acid concentration soybean genotypes with phosphorus starvation in hydroponics growth system. Appl. Biochem. Biotech..

[21] Fukuda Y., Tatsukawa E., Saneoka H., Hoshina T., Uefuji M., Raboy V. (2011). Growth characteristics, phytate concentrations, and coagulation properties of soymilk from a low-phytate Japanese soybean (*Glycine max* (L.) Merr.) line. Soil Sci. Plant Nutr..

[22] Sun J.S., Simpson R.J., Sands R. (1992). Nitrogenase activity of two genotypes of *Acacia mangium* as affected by phosphorus nutrition. Plant Soil.

[23] Naeem A., Aslam M., Lodhi A. (2018). Improved potassium nutrition retrieves phosphorus-induced decrease in zinc uptake and seed zinc concentration of wheat. J. Sci. Food Agric..

[24] Ao X., Guo X.H., Zhu Q., Zhang H.J., Wang H.Y., Ma Z.H., Han X.R., Zhao M.H., Xie F.T. (2014). Effect of phosphorus fertilization to P uptake and dry matter accumulation in soybean with different P efficiencies. J. Integr. Agric..

[25] Singh S.K., Reddy V.R., Fleisher D.H., Timlin D.J. (2018). Phosphorus nutrition affects temperature response of soybean growth and canopy photosynthesis. Front. Plant Sci..

[26] Wang J., Chen Y., Wang P., Li Y.S., Khan A. (2018). Leaf gas exchange, phosphorus uptake, growth and yield responses of cotton cultivars to different phosphorus rate. Photosynthetica.

[27] Ertl D., Young K.A., Raboy V. (1998). Plant genetic approaches to phosphorus management in agricultural production. J. Environ. Qual..

[28] Raboy V., Young K.A., Dorsch J.A., Cook A. (2001). Genetics and breeding of seed phosphorus and phytic acid. J. Plant Physiol..

[29] Meis S.J., Fehr W.R., Schnebly S.R. (2003). Seed source effect on field emergence of soybean lines with reduced phytate and raffinose saccharides. Crop. Sci..

[30] Oltmans S.E., Fehr W.R., Welke G.A., Raboy V., Peterson K.L. (2005). Agronomic and seed traits of soybean lines with low–phytate phosphorus. Crop. Sci..

[31] Wiggins S.J., Smallwood C.J., West D.R., Kopsell D.A., Sams C.E., Pantalone V.R. (2018). Agronomic Performance and Seed Inorganic Phosphorus Stability of Low-Phytate Soybean Line TN09-239. J. Am. Oil Chem. Soc..

[32] Singh S.K., Reddy V.R. (2016). Methods of mesophyll conductance estimation: Its impact on key biochemical parameters and photosynthetic limitations in phosphorus-stressed soybean across CO_2_. Physiol. Plant.

[33] Gillman J.D., Pantalone V.R., Bilyeu K. (2009). The low phytic acid phenotype in soybean line CX1834 is due to mutations in two homologs of the maize low phytic acid gene. Plant Genome.

[34] Panzeri D., Cassani E., Doria E., Tagliabue G., Forti L., Campion B., Bollini R., Brearley C.A., Pilu R., Nielsen E. (2011). A defective ABC transporter of the MRP family, responsible for the bean lpa1 mutation, affects the regulation of the phytic acid pathway, reduces seed myo-inositol and alters ABA sensitivity. New Phytol..

[35] Raboy V., Dickinson D.B. (1987). The timing and rate of phytic acid accumulation in developing soybean seeds. Plant Physiol..

[36] Oltmans S.E., Fehr W.R., Welke G.A., Raboy V., Peterson K.L., Peeler H.T. (1972). Biological availability of nutrients in feeds: Availability of major mineral ions. J. Anim. Sci..

[37] Abbasi M.K., Tahir M.M., Abbas W.A., Rahim N. (2012). Soybean yield and chemical composition in response to phosphorus-potassium nutrition in Kashmir. Agron. J..

[38] Yi X., Bellaloui N., MaClure A.M., Tyler D.D., Mengistu A. (2016). Phosphorus fertilization differentially influences fatty acids, protein, and oil in soybean. Am. J. Plant Sci..

[39] Krueger K., Goggi A.S., Mallarino A.P., Mullen R.E. (2013). Phosphorus and potassium fertilization effects on soybean seed quality and composition. Crop. Sci..

[40] Bethlenfalvay G.J., Schreiner R.P., Mihara K.L. (1997). Mycorrhizal fungi effects on nutrient composition and yield of soybean seeds. J. Plant Nutr..

[41] Toda K., Takahashi K., Ono T., Kitamura K., Nakamura Y. (2006). Variation in the phytic acid concentration of soybeans and its effect on consistency of tofu made from soybean cultivars with high protein concentration. J. Sci. Food Agric..

[42] Sánchez-Rodríguez A.R., del Campillo M.C., Torrent J. (2017). Phosphorus reduces the zinc concentration in cereals pot-grown on calcareous vertisols from southern Spain. J. Sci. Food Agric..

[43] Zhang Y.Q., Deng Y., Chen R.Y., Cui Z.L., Chen X.P., Yost R., Zhang F.S., Zou C.Q. (2012). The reduction in zinc concentration of wheat seed upon increased phosphorus-fertilization and its mitigation by foliar zinc application. Plant Soil.

[44] Zhang W., Liu D.Y., Li C., Chen X.P., Zou C.Q. (2017). Accumulation, partitioning, and bioavailability of micronutrients in summer maize as affected by phosphorus supply. Eur. J. Agron..

[45] Chen P.S.J., Toribara T.Y., Warner H. (1956). Microdetermination of phosphorus. Anal. Chem..

[46] Mariotti F., Tomé D., Mirand P.P. (2008). Converting nitrogen into protein-beyond 6.25 and Jones’ factors. Crit. Rev. Food Sci. Nutr..

[47] Raboy V., Dickinson D.B., Below F.E. (1984). Variation in seed total phosphorus, phytic acid, zinc, calcium, magnesium, and protein among lines of *Glycine max* and *G. soja* L.. Crop. Sci..

[48] Raboy V., Dickinson D.B. (1984). Effect of phosphorus and zinc nutrition on soybean seed phytic acid and zinc. Plant Physiol..

[49] Folch J., Lees M., Sloane Stanley G.H. (1957). A simple method for the isolation and purification of total lipids from animal tissues. J. Biol. Chem..

